# Angioedema in the Absence of C1 Esterase Inhibitor Deficiency in a Young Patient With Anti-dsDNA Negative Lupus Nephritis

**DOI:** 10.7759/cureus.39141

**Published:** 2023-05-17

**Authors:** Ifrah Nadeem, Dawlat Khan, Jiannan Huang, Sadia Aslam, Anum Nadeem, Wahab J Khan

**Affiliations:** 1 Internal Medicine, University of South Dakota Sanford School of Medicine, Sioux Falls, USA; 2 Internal Medicine, Avera McKennan Hospital and University Health Center, Sioux Falls, USA; 3 Physiology, Rashid Latif Medical College, Lahore, PAK

**Keywords:** c1 estrase inhibitor, pathophysiology, bradykinin mediated angioedema, angioedema, sle and lupus nephritis

## Abstract

Hereditary angioedema (HAE) is an autosomal dominant condition marked by a lack of functioning C1 esterase inhibitor (C1-INH). In contrast, acquired angioedema (AAE) due to a deficiency of C1 esterase inhibitor (AAE-C1-INH) may be the manifestation of an underlying lymphoproliferative, neoplastic, or autoimmune condition. Both are potentially fatal. The C1q protein is normal in HAE but low in AAE. A third mechanism has been reported to cause angioedema, especially in systemic lupus erythematosus (SLE) patients. AAE, which happens in association with SLE, may respond well to steroids. Here we present a case of AAE in a young female with SLE that led to upper airway compromise, requiring endotracheal intubation. Early detection and treatment of such cases can lead to an outstanding prognosis by preventing airway compromise and anoxic brain injury. Even though it is a condition of either very young or middle-aged patients, practitioners must be aware of this uncommon disease linked with SLE in adolescents and young adults.

## Introduction

Angioedema (AE) is a localized edema of the skin or mucosal surfaces which happens due to a localized inflammatory reaction leading to the extravasation of fluid into the interstitial compartment due to a loss of vascular integrity. An acquired uncommon condition of recurring angioedema episodes without urticaria that may or may not be associated with deficiency of C1 esterase inhibitor (C1-INH) is referred to as acquired angioedema (AAE) [1]. It constitutes 6-10% of all angioedema cases [2]. Swelling events both in AAE and hereditary angioedema (HAE) can be classified as either dermal, digestive, or upper airways. Patients with hereditary angioedema (HAE) are fairly healthy, whereas those with AAE-C1-INH may have a lymphoproliferative, malignant, or autoimmune disorder such as systemic lupus erythematosus (SLE) [3]. Laryngeal edema is a life-threatening condition that affects roughly half of AAE patients [4]. The exact pathogenesis is unknown, but it is thought to be caused by bradykinin, autoantibodies against the C1-INH protein, or activation of classical complement pathways [5]. The management strategy focuses on patient education about potential triggers as well as the treatment of the underlying condition [6].

## Case presentation

A 22-year-old female with a known history of hepatitis B e-antigen (HBeAg) negative chronic hepatitis B virus (HBV) infection presented with increasing bilateral lower extremity swelling, dark urine, and weight gain. Medical history was negative for associated fever, night sweats, skin rash, joint pain or swelling, hair loss, oral ulcers, photosensitivity, chest pain, shortness of breath, or abdominal pain. She denied tobacco, ethanol, or illicit drug use. The family history was negative for angioedema. She was not taking any over-the-counter or prescription medications. Her vital signs showed her afebrile with a blood pressure of 110/75 mm Hg, a heart rate of 91 beats per minute, and oxygen saturation of 96% on room air. Her physical exam was unremarkable except for bilateral lower extremities pitting edema up to the knees. Initial labs revealed a hemoglobin of 10.9 with a mean corpuscular volume (MCV) of 83 and a creatinine level of 2.1 mg/dL (n = 0.3-1.3 mg/dL), a C reactive protein (CRP) of 337 mg/L (n < 5 mg/L) and an albumin level of 2.8 g/dL (n = 3.3-4.4 g/dL). The rest of the labs consisting of platelets, white cell counts, electrolytes, and liver function tests, were normal. She was admitted for further workup of renal dysfunction. A renal ultrasound showed bilateral edematous kidneys with renal parenchymal hyperechogenicity and loss of normal corticomedullary differentiation without hydronephrosis. Advanced diagnostic workup revealed positive antinuclear antibodies-ANA (IFA titer of 1:360, speckled pattern) with a negative anti-double-stranded DNA (anti-dsDNA), anti-smith antibodies, cryoglobulins level, anti-neutrophilic cytoplasmic autoantibody (c and p-ANCA), and extractable nuclear antigen (ENA) panel. Complement studies showed hypocomplementemia with a C3 level of 37 mg/dL (n = 83-193 mg/dL) and a C4 level of 10.8 mg/dL (n = 15.0 - 57.0 mg/dL). Further lab work was also negative for cardiolipin immunoglobulin (Ig)G & IgM antibody, lupus inhibitor, beta-2 glycoprotein IgG & IgM, and dilute Russell viper venom time. Viral serology was negative for hepatitis C and HIV with positive hepatitis B surface antigen, hepatitis B core IgG, and positive hepatitis B e-antibodies. She underwent a renal biopsy that revealed exotoxin-associated membranous glomerulopathy and diffuse proliferative glomerulonephritis, an immune complex type specific for SLE nephritis.

**Figure 1:**
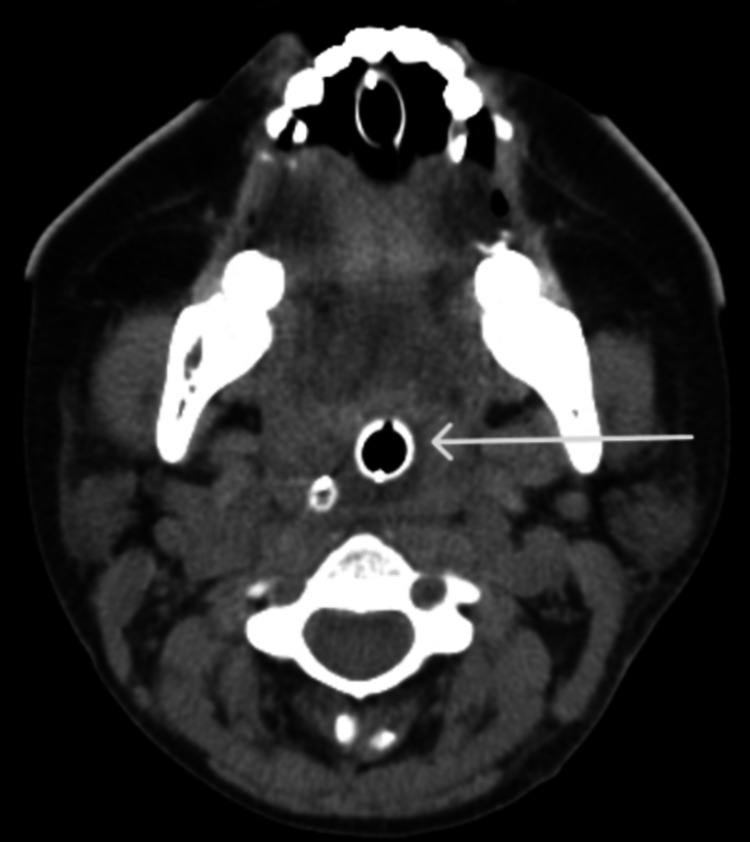
CT scan of the neck (axial section) The white arrow shows hypopharyngeal edema.

**Figure 2:**
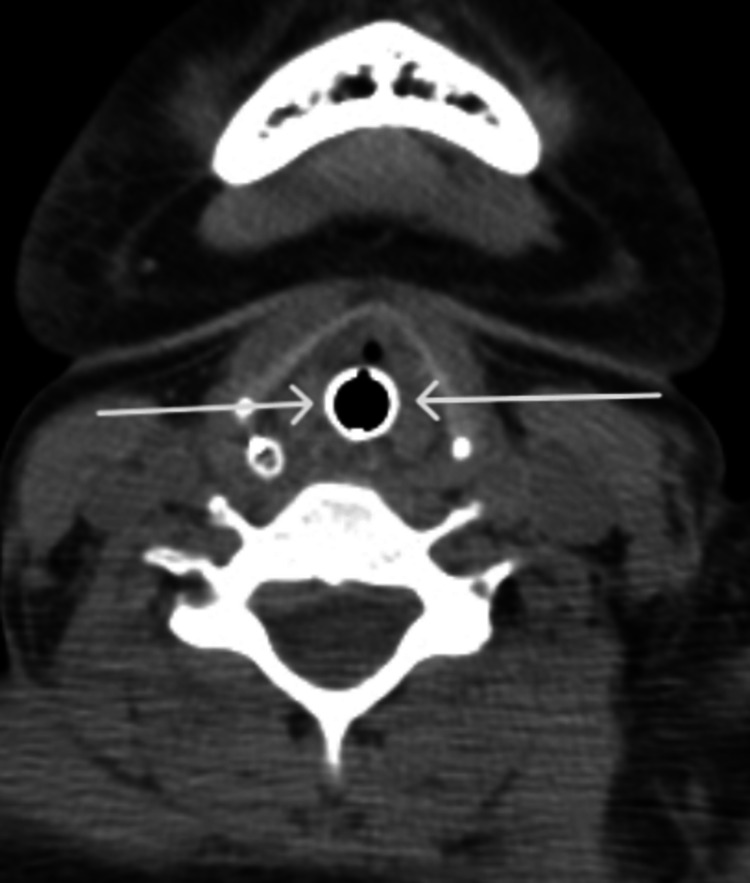
CT scan of the neck (axial section) White arrows show laryngeal edema at the level of true vocal cords.

She was started on mycophenolate mofetil, tacrolimus, oral steroids, and loop diuretics. On day-3 of admission, she reported throat swelling without associated lips swelling, pruritis, or hives. Vital signs showed signs of mild distress but no hypotension. CT neck revealed hypopharyngeal and retropharyngeal edema (Figure 1) and laryngeal edema at the level of true vocal cords (Figure 2).

Subsequently, she underwent a bedside flexible laryngoscopy assessment positive for significant epiglottis and arytenoid edema, followed by endotracheal intubation for airway protection. Further evaluation for angioedema showed normal C1 esterase inhibitors quantitative and functional assays with low C1q levels. She was started on high-dose intravenous steroids and continued immunosuppressive medications. Upon discharge, she continued immune suppressants with no further episodes of laryngeal edema on outpatient follow-up. A repeat C1q level after three months has normalized, and her renal function has been stable.

## Discussion

AE can broadly be classified into three types based on etiology and pathologic mechanism: mast cell-mediated, kinin-mediated, and unclassified. The first category is caused by mast cell degranulation and is usually associated with urticaria, wheezing, and hypotension. A trigger can usually be identified, typically an allergen causing a type-1 hypersensitivity reaction, radiocontrast media causing direct mast-cell degranulation, or a non-steroidal anti-inflammatory drug causing excessive leukotriene production through inhibition of cyclooxygenase. It can further be divided into idiopathic histaminergic (IH-AE) (responsive to antihistamine treatment) and idiopathic non-histaminergic (InH-AE) (non-responsive to antihistamines). On the other hand, kinin-mediated angioedema is characterized by angioedema in the absence of an identifiable trigger and without urticaria or other features of mast cell degranulation, as described above. Some examples of unclassified angioedema include angioedema related to drugs such as angiotensin-converting enzyme (ACE)-inhibitors (ACE-I-AAE) and fibrinolytic agents and urticarial vasculitis [7,8].

Kinin-mediated angioedema is caused by qualitative or quantitative deficiency of C1 esterase inhibitors which could be either hereditary or acquired. HEA is the most well-described form and is divided into type 1 (quantitative deficiency) or type 2 (qualitative deficiency), in which C1-INH is antigenically intact but nonfunctional. There is a third type of hereditary angioedema that occurs most commonly in women and is associated with many different mutations for proteins like Plasminogen and Angiopoietin-1. Although the pathogenesis is unclear, but is also believed to be associated with bradykinins, as evidenced by the response to a bradykinin B2 receptor antagonist icatibant [9]. AAE is due to an acquired deficiency of C1-INH (AAE-C1-INH). It less frequently causes abdominal symptoms and most commonly manifests in an older age compared to HEA, which is more common in a younger age group [10]. The majority of AAE cases since its description in the early 1970s have been found to be associated with a secondary disease process, with the most common being lymphoproliferative disorders and lymphomas (most often non-Hodgkin lymphomas and indolent lymphomas) [11]. The second most common association is autoimmune conditions. AAE has been reported in association with ulcerative colitis, myasthenia gravis, Sjogren syndrome, anti-phospholipid syndrome, anti-thrombin III deficiency, SLE, and immune thrombocytopenia [11]. AAE can be divided into type 1, also called paraneoplastic, associated with malignancies. This type is thought to develop due to direct activation and consumption of the classical complement pathway by lymphoproliferative neoplastic tissue [5,12]. Type 2, also called autoimmune acquired C1-INH deficiency, is defined by the presence of autoantibodies, and is mostly related to autoimmune diseases like SLE. The suggested mechanism behind the angioedema in these cases remains the bradykinin-associated extravasation of fluid into connective tissues. However, there have been reports of angioedema in lupus patients related to a third mechanism of major classical pathway- and alternative pathway-mediated complement consumption [13]. The levels of C4 and C2 are usually low, with a normal C3 level in cases of typical C1-INH deficiency leading to AAE. But a low C3 level shows major classical pathway-mediated complement consumption. Angioedema related to this mechanism is unusual but has been reported in the past, especially in association with SLE [13]. Further C1-INH levels and function may be normal in lupus patients presenting with AE [14]. This absence of C1-INH qualitative or quantitative dysfunction is similar to what has been ascribed to type 3 HEA or estrogen-dependent angioedema. In the past, it was postulated to be related to a transient rise in bradykinin itself without any pathologic modification in C1-INH antigenic function or level. Another explanation could be an imbalanced and compensatory increase in plasminogen synthesis, eventually leading to increased bradykinin levels transiently, especially in lupus nephritis patients with glomerulonephritis and resultant intermediate-size urinary protein loss. This hypothesis is further supported by the well-known tissue plasminogen activator (t-PA)-related angioedema cases. In-vitro plasmin and kallikrein (a precursor of bradykinin) make a synergistic positive feedback loop that can be inhibited by aminocaproic acid. This is likely the reason for tranexamic acid's effectiveness in certain AE cases. Another supportive point comes from the literature related to type 3 HAE. In these patients taking hormone replacement therapies (HRT) or oral contraceptive pills (OCP), plasmin and factors VII, X, and IX are increased, whereas plasminogen activator inhibitor is decreased. This is the same population that usually develops angioedema but has normal C1-INH levels and function [15]. Upper airway edema frequently involving the larynx is the most severe and potentially lethal site of angioedema. Around 50 % of patients with AAE-C1-INH experience upper airway edema and anoxic brain injury or death from upper airway obstruction [16].

Treatment of angioedema is most evident for the hereditary type. The therapeutic options depend upon the type, severity, and frequency of AE attacks. In acute phases, C1-INH concentrate, ecllantide, and icatibant have been well studied. In addition, androgens, C1-inhibitor concentrate, and fibrinolytic such as aminocaproic acid and tranexamic acid have been reported to be variably effective prophylactic agents [10]. Therapeutic options for AAE depend upon the underlying category. IH-AAE responds well to antihistamines, usually in higher-than-normal dosing. Antihistamines can also be used as prophylaxis in these patients. InH-AE has the least supported treatment evidence available in the literature, and there are a few case reports supporting tranexamic acid, steroids, and biological agents such as omalizumab use with variable success. This also reflects onto heterogeneity and complex pathophysiology of this particular type of AE [10]. ACE-I-AAE therapy involves avoiding the trigger, i.e., ACE-I permanently. Bradykinin-targeted drugs such as ecallantide and icatibant have shown a quick resolution of symptoms and early discharge from the hospitals [17]. In addition, angiotensin receptor blockers (ARBs) in these patients are not shown to increase the frequency or severity of recurrent attacks. Treatment of C1-INH AAE involves using bradykinin-targeted agents like HAE in addition to treating underlying diseases such as immunoproliferative disorders. There are a few case reports of monoclonal antibodies, such as rituximab use in this category, showing the reduction in frequency and severity of attacks as well as remission in some cases [10]. Treatment for AE in lupus patients, like its pathophysiology, is complex and not clear. Steroids have shown efficacy in multiple reports highlighting the primary pathologic driving force as the massive complement pathway activation likely related to underlying autoimmunity. Antihistamines have also been used in these cases successfully, indicating the interplay of mast cells and kinin pathways in this subset of angioedema patients [13,14].

Our patient presents a unique case from multiple aspects. She has no other SLE findings other than lupus nephritis and had negative anti-dsDNA and anti-smith antibodies. She presented at a relatively young age, whereas the typical onset of AAE is in the 4th decade of life [15]. She had normal C1-INH level and function with low C3 level, indicating a complex pathophysiology independent of C1-INH, likely a combination of major complement activation and transient rise in bradykinin levels. She responded well to steroids hinting at the mast-cell pathway's potential role. The limitation, in this case, is that no genetic testing was done for factor 12 or plasminogen that could categorize it in type 3 HAE or estrogen-dependent AE. But this entity seems less likely based on negative family history, no OCP/HRT use, and excellent response to steroids [10].

## Conclusions

AAE may happen in adolescents and young adults. It could be an early manifestation of SLE. The level and function of C1-INH may be normal in SLE patients who develop AE. The potential mechanism behind this type of angioedema is a complex interplay of both bradykinin and mast cell pathways and is likely related to massive complement activation and a transient rise in bradykinin levels.
